# Spatial transcriptomics and dual tracer mapping reveal Wnt-driven attenuation of blood-brain barrier permeability Induced by endothelial EphA4 deficiency

**DOI:** 10.1186/s40478-026-02394-9

**Published:** 2026-08-06

**Authors:** Caroline de Jager, Jing Ju, Marco Corbo, Karan Patel, Michelle H. Theus

**Affiliations:** 1 Department o f Biomedical Sciences and Pathobiology, Virginia Tech, 970 Washington Street SW, Life Sciences I; rm 249 (MC0910), Blacksburg, VA 24061 USA; 2 Signios Bio, Foster City, CA 94404 USA

**Keywords:** Traumatic brain injury (TBI), Eph signaling, Spatial transcriptomics, Brain microvasculature, Blood-brain barrier (BBB)

## Abstract

Blood-brain barrier (BBB) disruption is a key pathological event following traumatic brain injury (TBI), yet its molecular and spatial characteristics remain incompletely understood. Here, we developed a dual-tracer mapping system to assess the temporal and spatial dynamics of BBB permeability following controlled cortical impact (CCI) injury in *EphA4f/f VE-Cadherin-CreERT2* (KO) and *EphA4f/f* (WT) mice. By tracking Evans Blue Dye (EBD), sodium fluorescein (NaFl), and endogenous IgG deposition, we identified distinct patterns of vascular tracer extravasation in the injured cortex and hippocampus. NaFl, a small-molecule tracer, continued to extravasate for 7 days post-injury, whereas EBD leakage diminished after 4 days. Notably, endothelial EphA4 deletion significantly reduced size-dependent vascular tracer extravasation. To further investigate molecular pathways associated with altered BBB permeability, we integrated spatial transcriptomics with tracer quantification, revealing that endothelial EphA4 ablation upregulates BBB-associated genes (Tjp2, Tjp3, Cldn1, and Ocln) and injury-responsive genes linked to injury-responsive genes (Nr4a1 and Npas4). Wnt signaling genes were similarly upregulated in the KO cortex, and pharmacological inhibition of Frizzled-4 (FZD4)/Wnt signaling attenuated the reduction in vascular tracer extravasation observed in KO mice. Conversely, pharmacological activation of Wnt signaling with the FZD4 agonist FZM1.8 in WT mice reduced lesion volume and vascular tracer extravasation. Overall, these findings demonstrate the utility of combining spatial transcriptomics with dual-tracer mapping to identify region-specific transcriptional programs associated with BBB permeability following CCI injury and to define the contribution of EphA4/Wnt signaling to vascular responses after TBI. Together, these findings identify Wnt signaling as a promising therapeutic pathway for reducing vascular permeability and limiting secondary brain injury following TBI.

**Supplementary Information:**

The online version contains supplementary material available at https://doi.org/10.1186/s40478-026-02394-9.

## Introduction

Traumatic brain injury (TBI) is a leading cause of neurological dysfunction, with effects that extend well beyond the initial insult. Moderate to severe TBIs can lead to persistent neurological deficits and an increased risk of long-term complications [1]. Beyond the immediate consequences, secondary injury cascades, characterized by oxidative stress, neuroinflammation, and blood-brain barrier (BBB) disruption, exacerbate acute recovery and set the stage for long-term functional decline [2]. The BBB plays a critical role in maintaining brain homeostasis and is a primary target of injury-induced pathophysiological changes. Disruption of the BBB following controlled cortical impact (CCI) injury leads to increased vascular permeability, immune cell infiltration, and neuroinflammatory signaling, all of which contribute to secondary tissue damage. Despite its importance, the molecular mechanisms underlying BBB dysfunction following TBI remain poorly characterized, particularly in a spatially defined context.

Remarkable progress in describing cell states, both healthy and diseased, has been made using RNA sequencing approaches [3]. For example, single-cell RNA sequencing (scRNAseq) was first described in 2009 [4] and has enabled the exploration of cellular heterogeneity following TBI [5–7]. However, a key limitation of scRNA-seq is the loss of spatial resolution, which makes it difficult to capture the spatial organization of heterogeneous cell populations and molecular hallmarks in the injured brain. Addressing this gap, spatial transcriptomics provides a critical approach for mapping gene expression in situ, enabling a more comprehensive understanding of region-specific BBB dysfunction and cellular responses following TBI. Recent spatial transcriptomic studies have further demonstrated the utility of this approach for resolving region-specific molecular responses associated with acute TBI, highlighting the complexity and spatial heterogeneity of the injured neurovascular environment [8–10]. Understanding BBB disruption at high spatial resolution is essential for characterizing region-specific effects of TBI in preclinical animal models, enabling more rigorous design and assessment of BBB-targeted therapeutics [11, 12]. Localized assessments of BBB integrity may improve the mechanistic understanding of changes in vascular permeability that may drive regional disruptions of the injured milieu, including inflammation, neuronal function, and metabolic dysregulation, ultimately contributing to long-term neurological impairment [13].

Historically, BBB disruption has been studied using a number of tools, including Evans’ Blue Dye (EBD) absorption readings, quantitative PCR, and western blotting to probe expression changes, IgG deposition, and blood biomarkers (such as Occludin). However, these methods have significant limitations. EBD primarily binds to serum albumin, giving it a large molecular weight (~ 67 kDa) (PMID: 26578854) [14], which restricts its ability to detect subtle changes in BBB permeability. Additionally, chemical extraction methods used for EBD quantification can introduce variability by binding to proteins, thereby skewing absorbance readings [15]. Despite these challenges, there remains a critical need for improved methodologies that enable quantitative, region-specific assessments of BBB extravasation. Traditional techniques often lack spatial precision, making it difficult to map BBB permeability across heterogeneous brain regions following injury. To overcome these limitations, we developed a dual-dye mapping system using i.v. injection of sodium fluorescein (NaFl; <1 kDa) and EBD (69 kDa) alongside endogenous IgG staining (150 kDa), combined with spatial transcriptomics to quantify regional extravasation and gene expression changes at high resolution [16]. This study evaluated dual-dye and IgG extravasation during the acute phase of injury (1–7dpi).

Spatial sequencing provides a comprehensive view of the molecular landscape of the injured brain across different anatomical regions that display altered dye extravasation [17]. Importantly, we test this using KO mice, which our prior research has implicated as a key mediator of tissue damage and BBB disruption [18, 19]. Eph receptors and their ephrin ligands are bidirectional signaling partners [20] that have been linked to the CNS response to injury and neurodegenerative conditions [21]. By integrating spatial transcriptomics with functional tracer-based BBB permeability assays, this study provides a high-resolution molecular atlas of BBB permeability following TBI, highlighting EphA4 as a critical modulator of injury-induced vascular dysfunction and Wnt signaling as a promising therapeutic target for mitigating BBB integrity and secondary brain injury.

## Results

### Spatial and temporal quantification of BBB disruption in the CCI-Injured murine brain

To assess the temporal profile of BBB permeability following injury, wild-type (WT) mice underwent controlled cortical impact (CCI), and tracer extravasation into the injured cortex was quantified (Supplemental Fig. 1). One hour prior to tissue collection, mice received intravenous injections of Evans Blue Dye (EBD; albumin-bound, ~ 67 kDa) and sodium fluorescein (NaFl; 376 Da). Following circulation of the tracers, mice were transcardially perfused to remove intravascular blood and unbound dye, allowing subsequent measurements to reflect tracer extravasation across the BBB (Supplemental Fig. 1A).

Serial coronal sections were processed for IgG immunostaining (IgG, ~ 150 kDa) to further assess vascular permeability to endogenous blood-derived proteins. Confocal images were acquired (Supplemental Fig. 1B–H), and IgG, EBD, and NaFl extravasation was quantified. Representative images from 1-, 4-, and 7-days post-injury (dpi) show distinct temporal patterns of dye and protein extravasation (Supplemental Fig. 1F–H). At 1 dpi, extravasation of IgG, EBD, and NaFl was most pronounced, indicating substantial BBB disruption (Supplemental Fig. 1F). By 4 dpi, EBD and IgG extravasation were markedly reduced, whereas NaFl extravasation remained similar to that observed at 1 dpi (Supplemental Fig. 1G). By 7 dpi, EBD was fully excluded from the brain parenchyma, while NaFl extravasation persisted (Supplemental Fig. 1H). However, NaFl was no longer detected within the CCI-injured cortex by 14 dpi (Supplemental Fig. 1I–K). IgG immunoreactivity remained detectable at 7 dpi despite its larger molecular size relative to EBD, likely reflecting the persistence of previously extravasated endogenous protein within the injured parenchyma, whereas EBD measures vascular permeability only during the brief interval following tracer administration. Together, these data indicate that restoration of BBB permeability occurs in a size-dependent manner following CCI, with restriction of large macromolecular tracers preceding recovery of permeability restrictions to smaller solutes.

### Endothelial cell (EC)–specific EphA4 ablation reduces overall expression and alters temporal BBB dynamics after CCI-induced brain injury

**Fig. 1:**
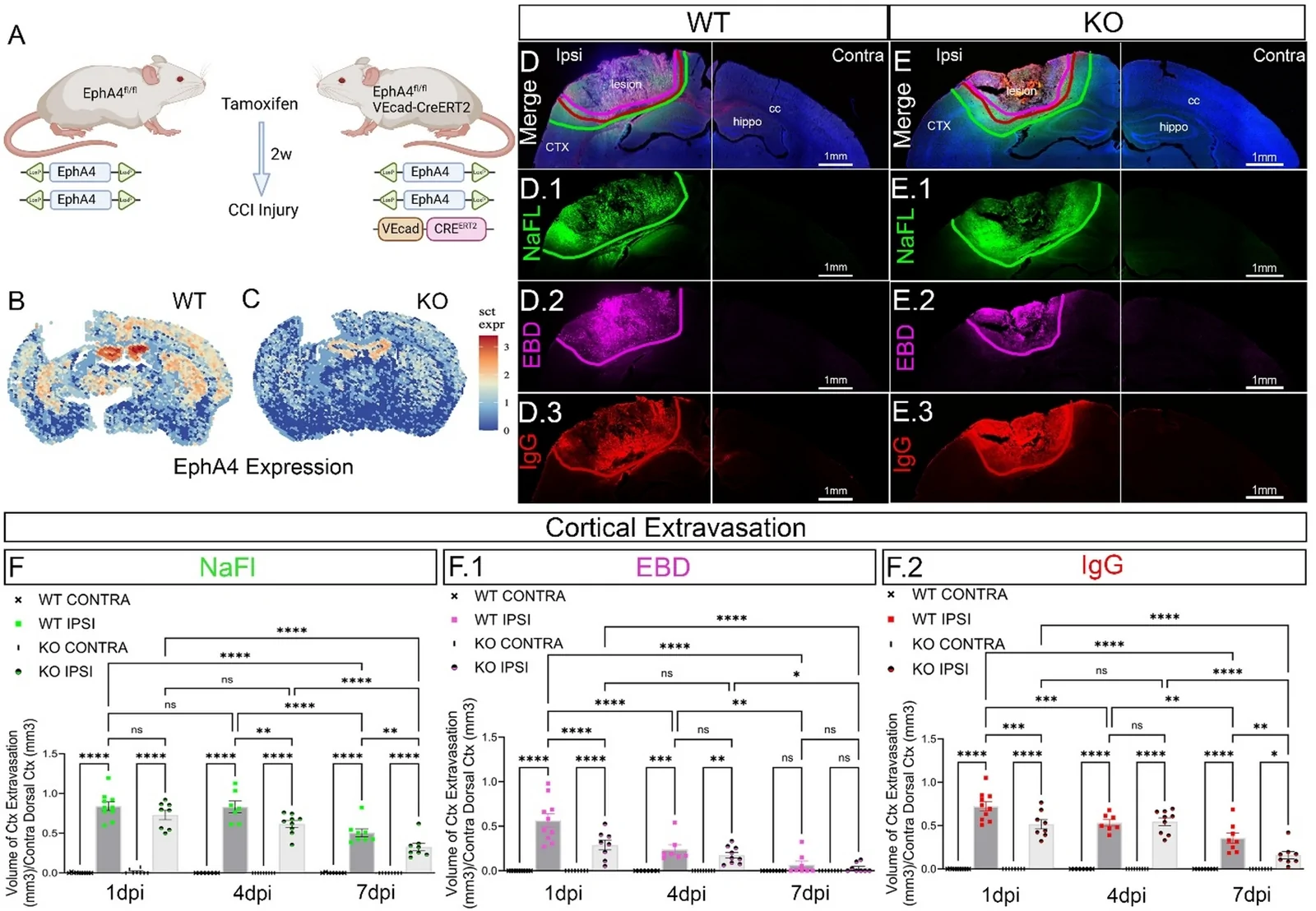
Endothelial cell–specific EphA4 ablation reduces overall expression and alters temporal BBB dynamics after CCI-induced brain injury. **A** Schematic representation of the genetic strategy used to generate KO mice. EphA4^fl/fl^ mice were crossed with VE-cadherin (VEcad)-CreERT2 mice to enable tamoxifen-inducible deletion of EphA4 in endothelial cells. Two weeks after tamoxifen administration, mice underwent CCI injury for subsequent analysis. (B–C) Spatial transcriptomic analysis of EphA4 expression in WT. **B** and KO. **C** brain sections following CCI injury. Heatmaps indicate relative EphA4 transcript levels, with warmer colors representing higher expression. Loss of endothelial EphA4 results in a marked reduction in overall EphA4 expression, particularly in the hippocampus, cortex, and thalamus. (D-D.3) Representative confocal images of cortical extravasation at 1dpi WT, showing merged signals **D**, NaFl (D.1, green), EBD (D.2, magenta), and IgG (D.3, red). − 1.6 A/P. (E-E.3) Representative confocal images of cortical extravasation at 1dpi KO, showing merged signals **E**, NaFl (E.1, green), EBD (E.2, magenta), and IgG (E.3, red). − 1.6 A/P. (F-F.2) Quantification of cortical extravasation of NaFl, **F**; EBD, (F.1); and IgG (F.2) at 1-, 4-, and 7-days post-injury (dpi) in WT and KO mice. Ipsilateral (IPSI) and contralateral (CONTRA) hemispheres are compared. Scale bar: 1 mm. *n* = 8–10. Statistical significance was determined using two-way ANOVA; Bonferroni post-hoc. Data presented as mean ± SEM; **p < 0.01, ***p < 0.001, ****p < 0.0001, ns = not significant

Endothelial EphA4 has previously been implicated in the pathogenesis of traumatic brain injury (TBI). To investigate its role in post-injury vascular dysfunction, *EphA4*^*f/f*^*/VE-Cadherin-Cre*^*ERT2*^ (KO) and *EphA4*^*f/f*^ (WT) mice were subjected to CCI injury. Adult male KO and WT mice received tamoxifen (50 mg/kg, i.p.) for five consecutive days to induce Cre-mediated recombination in endothelial cells. Following a 2-week washout period to allow recombination and clearance of tamoxifen metabolites, mice underwent CCI injury (Fig. 1A). To verify loss of EphA4 expression, spatial transcriptomic analysis (SpatialGE) was performed to visualize EphA4 transcript distribution across brain sections (Ospina et al., 2022, 2024, 2025). WT mice exhibited robust EphA4 expression throughout the injured hemisphere, including vascular-rich regions (Fig. 1B), whereas KO mice displayed markedly reduced EphA4 transcript levels (Fig. 1C), consistent with successful endothelial-targeted deletion. These data validate the inducible EC-specific EphA4 knockout model for investigating the contribution of endothelial EphA4 to BBB dysfunction and vascular permeability following TBI.

To assess the acute and subacute dynamics of BBB permeability, we quantified EBD, NaFl, and IgG extravasation in the contralateral (CONTRA) and ipsilateral (IPSI) cortices of WT and KO mice at 1-, 4-, and 7-days post-injury (dpi) (Fig. 1D and F.2). NaFl extravasation was consistently greater than EBD extravasation at all time points, reflecting size-dependent differences in BBB permeability (Supplemental Fig. 2). EBD extravasation was significantly elevated in the injured cortex at 1 dpi and 4 dpi but returned to near-baseline levels by 7 dpi, indicating progressive restoration of BBB integrity to large circulating macromolecules (Fig. 1E.1). In contrast, NaFl extravasation remained elevated at 7 dpi, suggesting sustained permeability to small molecular tracers (Fig. 1E). IgG immunoreactivity exhibited a similar temporal pattern, with a marked reduction between 1 dpi and 4 dpi and no further decrease at 7 dpi (Fig. 1E.2). The persistence of IgG signal at later time points likely reflects retention of previously extravasated endogenous protein within the injured parenchyma rather than ongoing vascular leakage. As expected, little to no extravasation was observed in the contralateral cortex across all tracers and time points (Fig. 1D and E.2).

Given the differential permeability of large and small tracers across the post-injury period, we next examined whether endothelial EphA4 deletion altered BBB recovery. Dye and protein extravasation were quantified in EphA4 KO and WT mice to determine the contribution of endothelial EphA4 to BBB dysfunction following CCI (Fig. 1E–E.2). NaFl extravasation was significantly reduced in KO cortices at 4 dpi and 7 dpi compared with WT mice, indicating improved barrier function to small molecular tracers during the subacute phase of injury (Fig. 1E). No genotype differences were observed at 1 dpi, suggesting comparable early permeability to small solutes. In contrast, EBD extravasation was significantly reduced in KO mice at 1 dpi, while IgG deposition was reduced at both 1 dpi and 7 dpi (Fig. 1E.1–E.2). Together, these findings identify endothelial EphA4 as a key regulator of BBB dysfunction following TBI and suggest that its loss both attenuates acute vascular disruption while promoting restoration of barrier integrity.

Although the primary lesion was cortical, dye and protein extravasation extended into the hippocampus, where regional differences between WT and KO mice were also observed (Supplemental Fig. 3A–C.2). This finding is of particular interest given the well-established vulnerability of hippocampal circuitry to secondary injury and cognitive dysfunction following TBI [22–25]. EBD extravasation increased in WT tissue between 4 and 7 dpi but did not differ between WT and KO mice (Supplemental Fig. 3C.1). Similarly, IgG deposition decreased between 1 and 4 dpi in WT hippocampi but showed no genotype-dependent differences (Supplemental Fig. 3C.2). In contrast, NaFl extravasation was significantly reduced in KO tissue compared with WT at 1 dpi and 4 dpi, although this difference was no longer evident by 7 dpi (Supplemental Fig. 3C). Representative images of hippocampal tracer extravasation at 1 dpi illustrate these genotype-dependent differences (Supplemental Fig. 3A.1–A.4 and B.1–B.4). Together, these findings indicate that the protective effects of endothelial EphA4 deletion extend beyond the primary cortical lesion and are associated with reduced hippocampal permeability to small molecular tracers following TBI.

###  Spatial transcriptomics reveals EphA4-dependent gene expression changes and pathway enrichment in the injured cortex

**Fig. 2:**
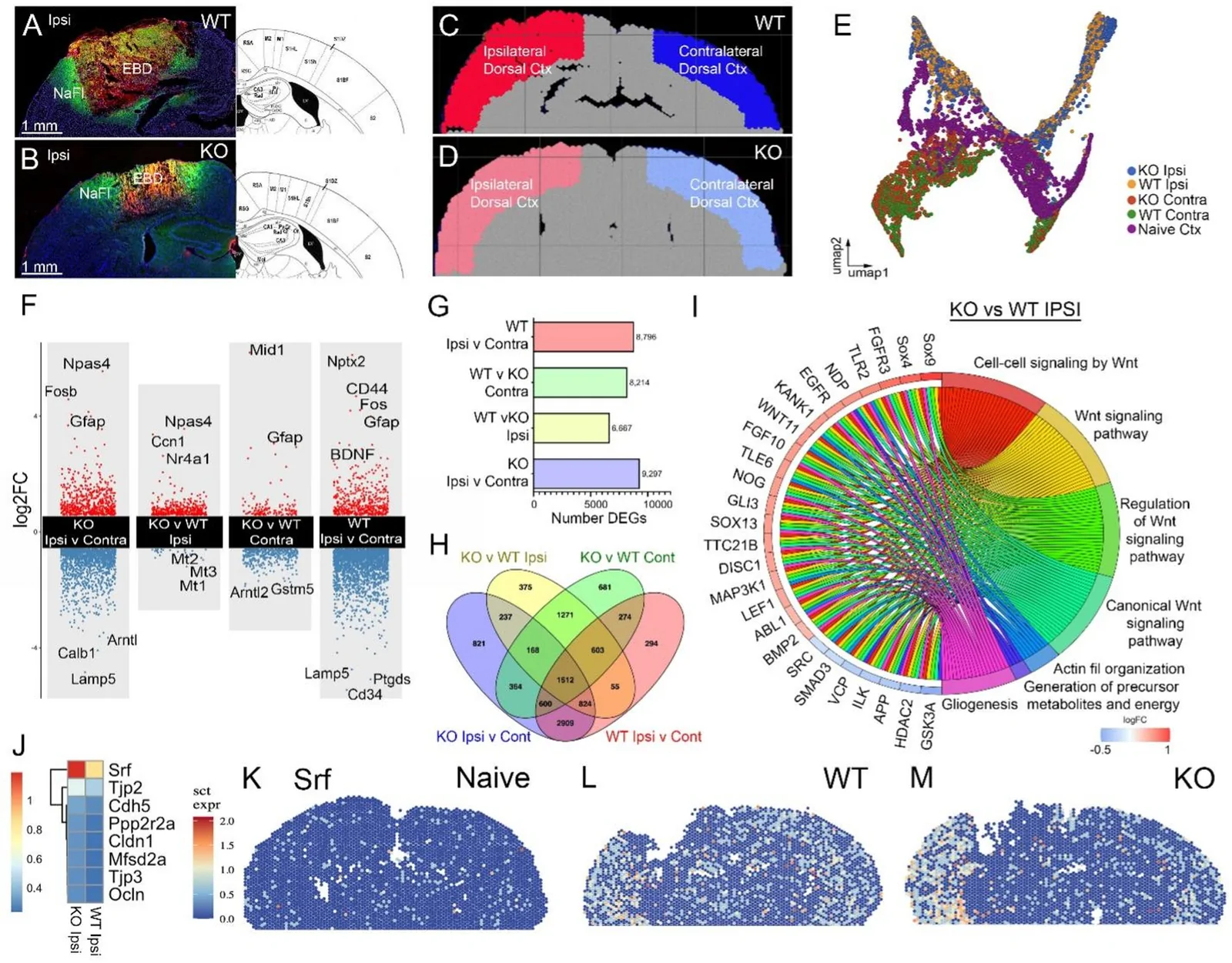
Comparative spatial sequencing analysis of the damaged cortex in WT and KO mice 1 dpi. **A**, **B**) Ipsilateral coronal sections of the brain from WT and KO mice, respectively, showing a representative confocal image of EBD (magenta), NaFl (green), and DAPI (blue). The images illustrate differences in dye extravasation between WT and KO mice. − 1.2 A/P. **C**, **D** Annotated clusters of the ipsilateral and contralateral somatosensory cortex (SSC) in the WT and KO dorsal cortex, respectively. **E** UMAP showing clustering of spatial sequencing data from different brain regions in WT and KO mice. Each color represents a different region: KO Ipsilateral cortex (blue), KO Contralateral cortex (red), WT Ipsilateral cortex (yellow), WT Contralateral cortex (green). **F** Transformed volcano plot showing differentially expressed genes between different cortical regions, highlighting significant genes such as *Gfap*, *Bdnf*, *Mid1*, and *Npas4*. **G** Bar graph displaying the number of differentially expressed genes across cortical regions and conditions. **H** Venn diagram illustrating the overlap of differentially expressed genes among four cortical comparisons between WT and KO. **I** Circular chord plot depicting signaling pathways enriched in differentially expressed genes in KO versus WT Ipsilateral cortex, revealing the Wnt signaling pathway enriched in KO cortex. **J** Heatmap of genes important for BBB function and integrity. **K**–**M** Feature plots of Srf gene expression in the brain tissue of Naïve WT (**K**), WT CCI (**L**), and KO CCI (**M**) mice

To characterize transcriptional changes associated with cortical injury, we performed Visium spatial transcriptomic analysis (10x Genomics) on brain sections collected 1 day post-injury (dpi) from WT and KO mice. Regions of interest (ROIs) within the dorsal cortex, excluding the motor cortex, were identified and analyzed using the Partek classification workflow (Fig. 2A and D). Uniform Manifold Approximation and Projection (UMAP) analysis revealed distinct clustering of naïve, injured, and adjacent uninjured cortical regions, indicating robust transcriptional segregation based on injury status (Fig. 2E). Consistent with previous reports, differential gene expression analysis of WT tissue demonstrated significant upregulation of canonical injury-associated genes, including *Fos*,* Gfap*,* Cd44*, and *Bdnf*, within the injured cortex relative to uninjured regions (Fig. 2F), reflecting activation of inflammatory, glial, and injury-responsive signaling pathways [26–28].

To determine how endothelial EphA4 influences the cortical injury response, we compared transcriptional profiles between WT and KO injured cortex (Fig. 2F and H). Differential gene expression analysis identified several injury-responsive genes that were significantly increased in KO tissue, including *Npas4* and *Nr4a1* (Fig. 2F) [29, 30]. Conversely, expression of the metallothionein family members *Mt1*,* Mt2*, and *Mt3*, which are commonly induced during oxidative stress responses, was reduced in KO tissue (Fig. 2F). Across all pairwise comparisons, the number of differentially expressed genes (DEGs) ranged from 6667 to 9297 (Fig. 2G), with 1512 DEGs shared among all four comparisons, indicating both conserved injury-associated transcriptional programs and EphA4-dependent molecular responses (Fig. 2H).

Gene ontology (GO) enrichment analysis of the KO versus WT injured cortex identified significant enrichment of pathways associated with Wnt signaling, actin filament organization, gliogenesis, and metabolic precursor generation (Fig. 2I), suggesting that endothelial EphA4 deletion alters multiple molecular programs involved in tissue repair and recovery following injury. Consistent with the improved BBB integrity observed in KO mice, a focused heatmap of BBB-associated genes revealed increased expression of several transcripts critical for endothelial barrier function, including *Cldn1*,* Ocln*,* Mfsd2a*,* Tjp2*, and *Tjp3* (Fig. 2J). Among these, *Serum response factor (Srf)*, a transcription factor known to regulate endothelial stability and BBB integrity, was significantly upregulated in the KO injured cortex relative to WT injured tissue (Fig. 2L and M) [31]. Notably, both WT and KO injured cortices exhibited higher *Srf* expression than naïve WT cortex (Fig. 2K), suggesting that injury induces *Srf* expression, which is further enhanced by endothelial EphA4 deletion.

### Spatial transcriptomics reveals region-specific gene expression changes following BBB disruption and EC-specific EphA4 deletion

**Fig. 3:**
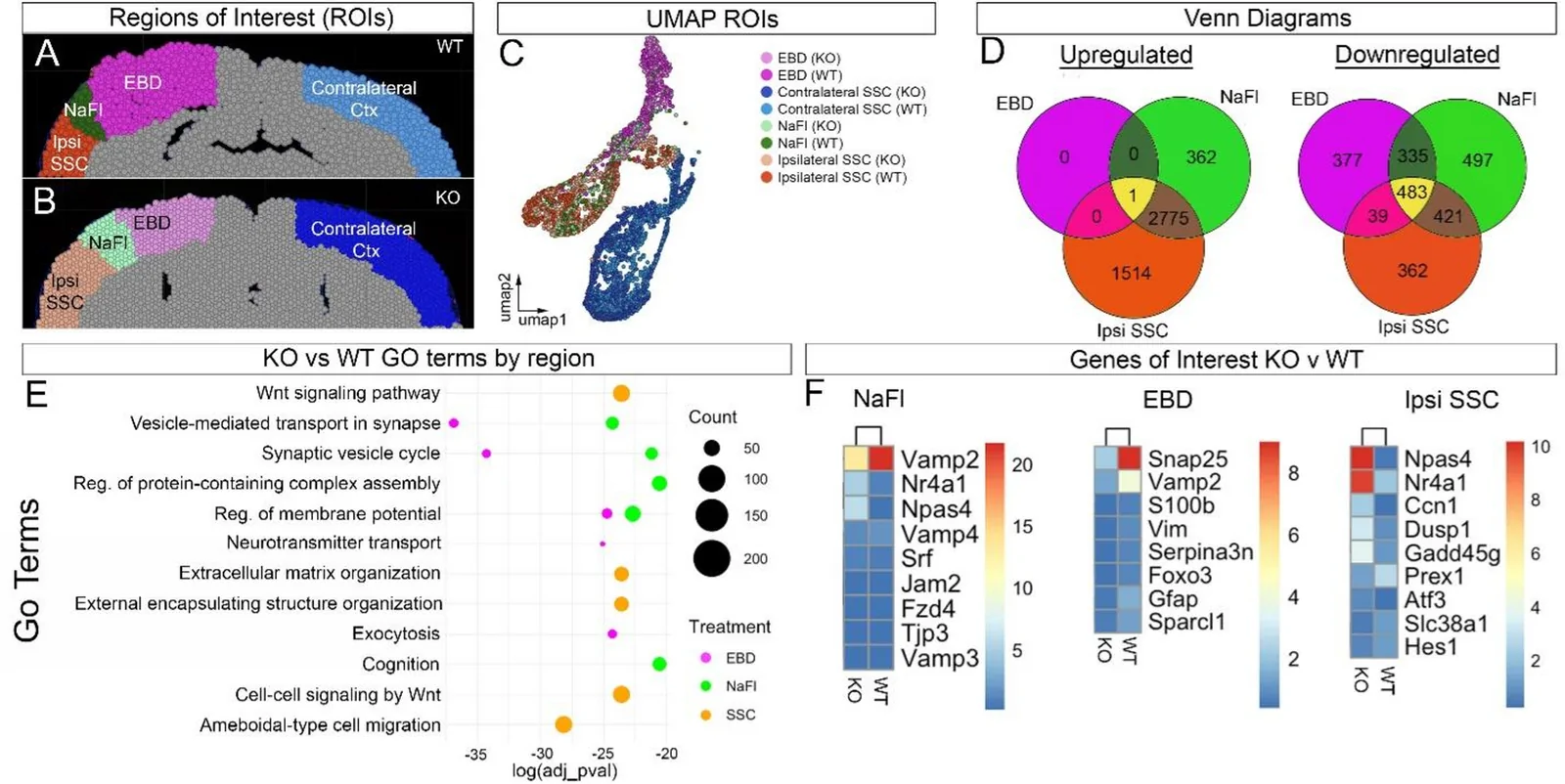
Spatial and molecular heterogeneity of transcriptomic changes across dye-labeled regions in EC-specific EphA4 KO mice. **A**, **B** Regions of Interest (ROIs) for EBD and NaFl in the brain are shown of KO and WT CCI-injured mice. The regions include EBD and NaFl-stained ipsilateral cortex, contralateral somatosensory cortex (Contralateral SSC), and ipsilateral somatosensory cortex (Ipsi SSC). − 1.2 A/P. **C** UMAP displaying the clustering of different ROIs based on gene expression profiles obtained through spatial sequencing. Each color represents a different ROI for each genotype: Naïve, EBD, NaFl, contralateral SSC, and Ipsi SSC. **D** Venn diagrams illustrating the number of upregulated and downregulated genes in each ROI when comparing KO to WT mice. **E** Gene Ontology (GO) enrichment analysis comparing KO to WT across EBD, NaFl, and SSC brain regions. The dot plot indicates significant GO terms related to biological processes, including vesicle-mediated transport in the synapse, regulation of the postsynaptic membrane potential, and others. Dot size represents the number of genes from the input list that are annotated to a given GO, while color indicates the brain region. **F** Heatmaps showing specific DEGs of interest in KO and WT mice across three ROIs: NaFl, EBD, and Ipsi SSC. Notable genes include Vamp2, Npas4, Nr4a1, Foxo3, and Hes1, among others, which regulate transcytosis

To determine whether distinct levels of BBB disruption are associated with unique transcriptional responses, we compared spatial gene expression profiles across cortical regions defined by vascular tracer extravasation and injury status (Fig. 3A and B). Regions of interest (ROIs) corresponding to EBD-positive, NaFl-positive, ipsilateral tracer-negative, and contralateral uninjured cortex were identified and analyzed by spatial transcriptomics. Uniform Manifold Approximation and Projection (UMAP) analysis revealed clear segregation of these regions, indicating distinct transcriptional programs associated with varying degrees of BBB permeability and tissue injury (Fig. 3C). Differential expression analysis further identified both shared and region-specific transcriptional responses, with Venn diagram analysis demonstrating substantial overlap as well as unique sets of differentially expressed genes (DEGs) between WT and KO tissues across the defined cortical regions (Fig. 3D).

To further characterize molecular pathways altered by endothelial EphA4 deletion, we performed GO analysis of DEGs identified within the EBD- and NaFl-defined regions. GO enrichment revealed distinct biological processes associated with each permeability-defined ROI, including post-synaptic organization, regulation of membrane potential, and extracellular matrix organization (Fig. 3E), indicating that spatially distinct regions of BBB disruption are associated with unique transcriptional responses following injury.

Differential expression analysis further identified several genes that were selectively altered in KO tissue across individual ROIs (Fig. 3F). Within the NaFl-positive region, the vesicle trafficking genes *Vamp2* and *Vamp4*, which have been implicated in transendothelial transport processes, were downregulated, whereas *Nr4a1*, an injury-responsive transcription factor implicated in cellular adaptation to stress, was significantly upregulated. Within the EBD-positive region, expression of *Vim* and *Gfap*, canonical markers of reactive astrocytes, was reduced in KO tissue, suggesting attenuated astrocytic activation following injury. In the ipsilateral somatosensory cortex, *Nr4a1* expression was similarly elevated, indicating a consistent association between endothelial EphA4 deletion and activation of injury-responsive programs. Together, these findings demonstrate that permeability-defined regions exhibit distinct molecular signatures following CCI and suggest that endothelial EphA4 deletion modulates region-specific pathways involved in neurovascular remodeling, glial activation, and tissue repair.

### EC-specific EphA4 deletion alters regional dye-specific gene expression and highlights synaptic pathways following CCI injury

**Fig. 4:**
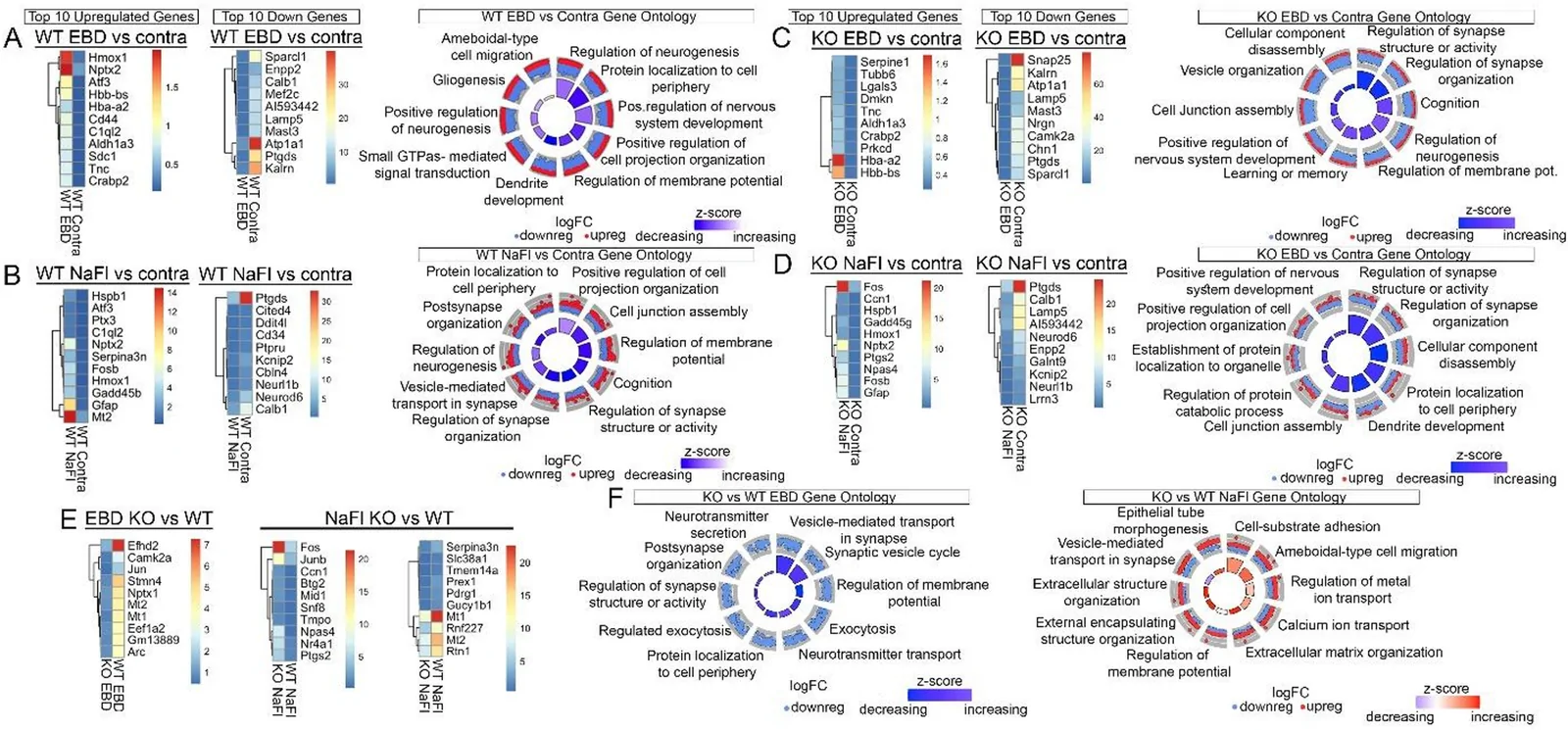
Top genes and comparative gene ontology (GO) analysis across dye regions in KO compared to WT injured mice. **A** GO enrichment analysis comparing EBD vs. control, highlighting significant biological processes and pathways affected. **B** GO enrichment analysis comparing NaFl vs. control, showing the impact on various cellular functions. **C** KO EBD vs. control GO ontology, illustrating the differential gene expression and associated biological processes. **D** KO NaFl vs. control GO ontology, depicting changes in gene expression and relevant biological pathways. **E** KO vs. WT EBD and NaFl comparison, indicating significant alterations in gene function between knockout and wild-type samples. **F** KO vs. WT EBD and NaFl GO ontology, summarizing the differences in gene expression and functional categories

To characterize transcriptional differences associated with varying degrees of BBB disruption, we analyzed differentially expressed genes (DEGs) and enriched pathways within EBD- and NaFl-defined cortical regions relative to contralateral tissue in WT and KO mice (Fig. 4A and D). Within WT EBD-positive regions, genes associated with hemorrhage-associated transcripts and vascular injury (*Hbb-bs* and *Hba-a2*) were significantly upregulated, whereas genes involved in synaptic transmission and neuronal homeostasis (*Sparcl1* and *Ptgs2*) were reduced (Fig. 4A). Consistent with these transcriptional changes, Gene Ontology (GO) analysis identified enrichment of pathways related to neurogenesis and neuronal signal transduction, suggesting impaired neuronal function within regions exhibiting severe BBB disruption (Fig. 4A). In contrast, WT NaFl-positive regions were characterized by increased expression of injury-responsive and inflammatory genes, including *Gfap*,* Ptx3*,* Atf3*, and *Fosb*, together with reduced expression of genes associated with neuronal plasticity and memory, including *Calb1* (Fig. 4B). GO enrichment analysis further revealed enrichment of pathways involved in synaptic organization and neurogenesis (Fig. 4B), indicating that even regions exhibiting selective permeability to small molecular tracers undergo substantial transcriptional remodeling following injury [32].

Likewise, KO EBD regions exhibited a transcriptional profile similar to that observed in WT EBD regions, characterized by increased expression of hemoglobin-associated genes (*Hba-a2* and *Hbb-bs*) together with genes involved in extracellular matrix remodeling and reactive injury responses, including *Serpine1*,* Lgals3*, and *Tnc* (Fig. 4C). These findings suggest that EBD-positive regions represent a vascular injury core enriched for blood-derived products and tissue remodeling processes following CCI. Concurrently, genes involved in synaptic transmission and neuronal signaling, including *Snap25*,* Camk2a*, and *Sparcl1*, were reduced relative to contralateral tissue (Fig. 4C).

In KO NaFl regions, we observed increased expression of genes associated with astrocyte activation (*Gfap*) and activity-dependent transcriptional responses (*Fos*,* Npas4*, and *Gadd45g*), alongside reduced expression of genes linked to neuronal plasticity and memory function (*Calb1* and *Lamp5*) (Fig. 4D). Consistent with these findings, GO analysis identified enrichment of pathways involved in synaptic organization, dendrite development, cell-junction assembly, and regulation of membrane potential (Fig. 4D).

Direct comparison of KO and WT EBD regions revealed reduced expression of genes involved in cytoskeletal dynamics, vesicular transport, calcium signaling, and neuronal function, including *Efhd2*,* Camk2a*,* Stmn4*, and *Nptx1* (Fig. 4E). GO enrichment analysis highlighted pathways related to synapse structure and activity, synaptic vesicle function, neurotransmitter secretion, and membrane potential regulation (Fig. 4F), suggesting altered neuronal signaling within the EBD-defined injury core. In contrast, KO NaFl regions exhibited increased expression of *Fos*,* Junb*,* Npas4*, and *Nr4a1*, genes implicated in activity-dependent plasticity and injury adaptation while transcripts associated with neuroinflammatory and GABAergic signaling pathways, including *Serpina3n* and *Slc38a1*, were reduced (Fig. 4E). Gene ontology analysis further demonstrated enrichment of pathways involved in extracellular matrix organization and regulation of membrane potential, accompanied by reduced vesicle-mediated transport at the synapse (Fig. 4F). Together, these findings demonstrate that regions exhibiting distinct levels of BBB disruption possess unique transcriptional signatures and suggest that endothelial EphA4 deletion promotes molecular programs associated with synaptic maintenance, and neurovascular remodeling following CCI injury.

### Spatial transcriptomics identifies Wnt signaling as a candidate mediator of BBB permeability following endothelial EphA4 deletion

**Fig. 5:**
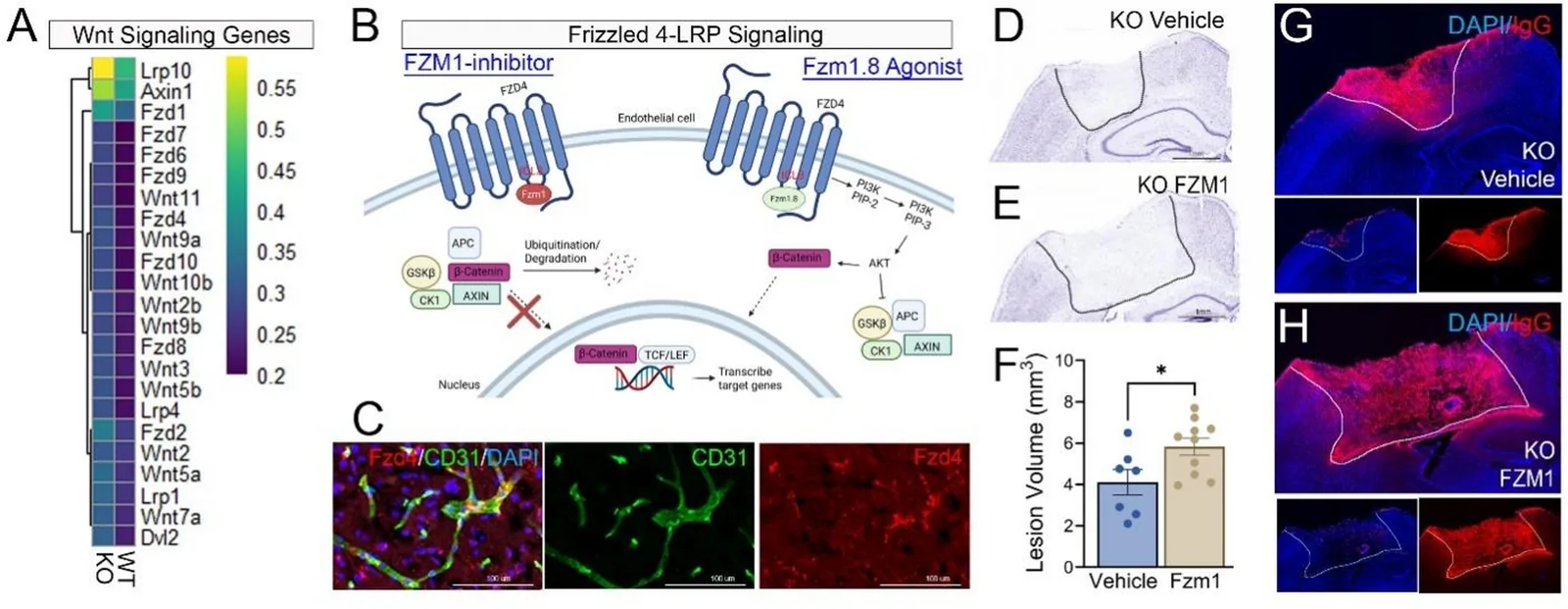
Modulation of Wnt signaling in EC-specific EphA4 KO endothelium influences BBB integrity and lesion volume following CCI injury. **A** Heatmap of Wnt signaling gene expression highlighting differential expression of key components in WT and KO ipsilateral cortex. Color intensity corresponds to relative expression levels. **B** Schematic representation of Fizzled-4 (FZD4)–LRP5/6 signaling pathway, and associated downstream effectors (e.g., GSK3β, β-catenin). **C** Representative max-z projection confocal image of immunofluorescence showing Fzd4 (red) and CD31 (green) co-localization in brain vasculature, indicating endothelial expression of Wnt-related signaling components. **D**, **E** Nissl-stained brain tissue from KO mice treated with vehicle or FZD4 inhibitor, FZM1. Quantification in **F** reveals a significant increase in lesion volume in FZM1-treated KO mice (*n* = 7–10, t-test; *p* < 0.05). **G**–**H** IgG extravasation in KO mice treated with vehicle (**G**) or FZM1 (**H**). Dashed lines delineate regions of IgG leakage, indicative of BBB disruption. − 1.6 A/P

Spatial transcriptomic analysis revealed increased expression of multiple Wnt signaling components within the KO ipsilateral cortex compared to WT injured tissue, including *Wnt7a*,* Wnt9a*,* Wnt10b*,* Fzd1*,* Fzd4*, and *Lrp1* (Fig. 5A), suggesting enhanced activation of Wnt-associated signaling pathways following endothelial EphA4 deletion. Given the established role of Wnt signaling in regulating BBB development and maintenance [33–35], we next investigated whether activation of this pathway contributes to the improved vascular phenotype observed in KO mice. Fzd4, a key receptor involved in endothelial Wnt/β-catenin signaling, was predominantly localized to CD31 + cerebral blood vessels within the injured cortex (Fig. 5C), supporting a potential role for endothelial Wnt signaling in BBB permeability. A schematic illustrating Frizzled4-LRP-mediated activation of canonical Wnt signaling is shown in Fig. 5B.

To determine whether enhanced Wnt signaling contributes to acute BBB stabilization in EC-specific EphA4 KO mice, we pharmacologically inhibited Frizzled4 (Fzd4)-mediated Wnt signaling using the allosteric Fzd4 antagonist Fzm1 at 1 dpi, a time point corresponding to maximal BBB disruption following CCI. Inhibition of Fzd4 signaling significantly increased lesion volume and IgG extravasation in KO mice compared with vehicle-treated controls (Fig. 5D and H), indicating loss of the BBB-protective phenotype associated with endothelial EphA4 deletion.

**Fig. 6:**
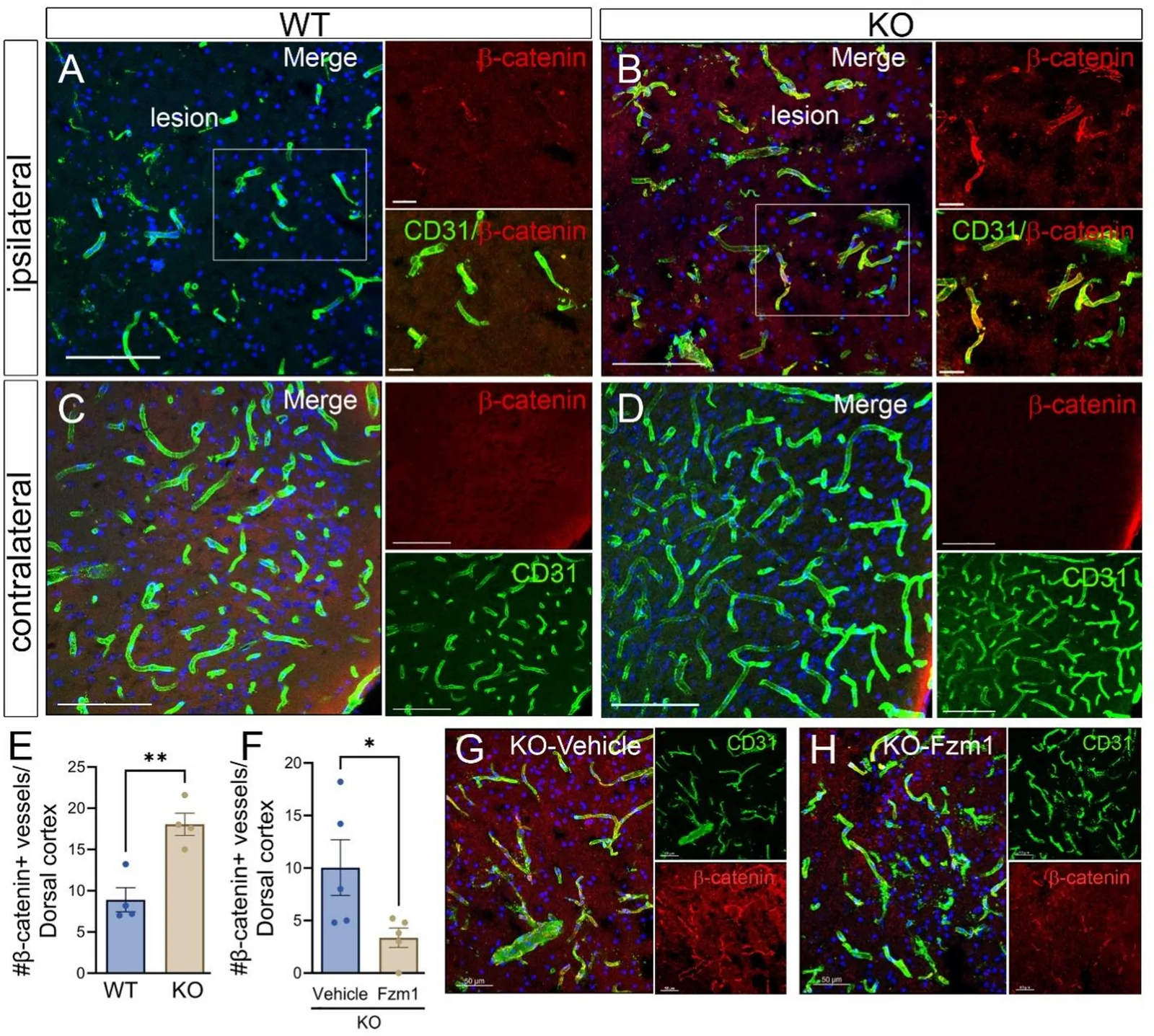
Loss of endothelial cell (EC) EphA4 increases vascular β-catenin expression, which is attenuated using Frizzled 4 (FZD4) receptor inhibitor Fzm1 after 1-day CCI injury. **A**–**C** Representative confocal images of CD31+ (green) and β-catenin+ (red) vessels in the WT ipsilateral (**A**), KO ipsilateral (**B**), and WT contralateral (**C**) and KO contralateral cortices at 1 dpi. Images represent selected fields from the injured cortex and were used to assess vascular β-catenin expression within CD31 + blood vessels. Apparent differences in vessel number between representative images reflect regional variation and field selection and were not used to assess vascular density. **E** Quantification of β-catenin+/CD31 + vessels in the WT and KO ipsilateral cortex. **F** Quantification of β-catenin+/CD31 + vessels in the KO ipsilateral cortex after vehicle **G** or Fzm1 **H** treatment at 1 dpi. Contralateral images are shown as baseline controls but were not included in the quantitative comparison because vascular β-catenin signal was minimal to absent. Statistical significance was determined using an unpaired t-test. Data presented as mean ± SEM; T-test in D and E. *n* = 4–5/group. *p < 0.05, **p < 0.01. Scale=100 μm in A–D; 50 μm in G–H

To confirm modulation of canonical Wnt signaling, we quantified vascular β-catenin expression within the injured cortex. Ipsilateral vascular β-catenin expression was significantly increased in KO (Fig. 6B and E), relative to WT control mice (Fig. 6A and E), consistent with enhanced activation of Wnt signaling following EphA4 deletion. In contrast, Fzm1 treatment attenuated vascular β-catenin expression (Fig. 6H and F) compared to vehicle treatment (Fig. 6G and F) in KO mice, confirming effective suppression of Wnt/β-catenin signaling. No vascular expression of β-catenin is observed in the contralateral, non-injured cortex (Fig. 6C and D).

**Fig. 7:**
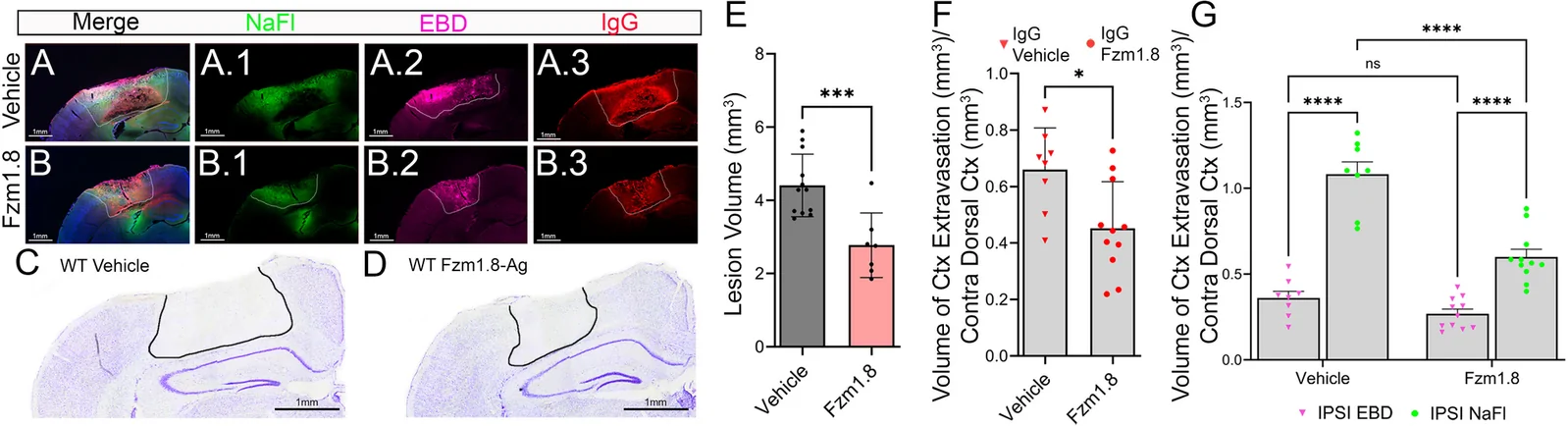
Wnt signaling activation using Frizzled 4 (FZD4) receptor agonist protects BBB integrity and reduces lesion volume. (A-A.3; B-B.3) Effects of FZD4 agonist (Fzm1.8) on BBB permeability. Merged images and separate channels show NaFl (green), EBD (magenta), and IgG (red), showing BBB impairment under vehicle conditions and protection with Wnt activation. **F**–**G** Quantification of extravasation volume in vehicle control versus Fzm1.8-treated groups, showing significantly reduced NaFl and IgG leakage with Wnt agonists (*n* = 8–12, two-way ANOVA; ****p < 0.0001, *p < 0.05, ns = not significant). **C**–**E** Lesion volume quantification showing a significant decrease with Fzm1.8 treatment compared to control (*n* = 7–11; unpaired t-test, ***p < 0.001). (**C**–**D**) Representative Nissl images (− 2.4 A/P), corresponding to quantitative measures in (**E**)

We next examined whether direct activation of endothelial Wnt signaling could reduce vascular permeability following TBI. WT mice were treated with the Frizzled4 agonist Fzm1.8, an allosteric activator of Fzd4-dependent Wnt/β-catenin signaling [36]. Because Fzd4 expression was largely restricted to CD31 + endothelial cells within the injured cortex (Fig. 5C), Fzm1.8 provided a means to selectively enhance endothelial Wnt signaling. Fzm1.8 treatment significantly reduced NaFl and IgG extravasation compared with vehicle-treated controls (Fig. 7A–B and F–G), indicating reduced vascular permeability. In addition, lesion volume was significantly reduced at 1 dpi in Fzm1.8-treated mice (Fig. 7C and E), demonstrating protection against secondary tissue damage. Together, these findings establish Wnt/β-catenin signaling as a critical downstream mediator of endothelial EphA4 function following TBI. Pharmacological inhibition of Fzd4 signaling abolished the protective phenotype observed in EphA4-deficient mice, whereas activation of endothelial Wnt signaling attenuated vascular tracer extravasation and reduced lesion volume. These results identify endothelial EphA4 as a negative regulator of Wnt-dependent vascular barrier stabilization in the acute phase following injury and support therapeutic targeting of the Wnt/Fzd4 pathway to promote vascular recovery following traumatic brain injury.

## Discussion

These findings provide new insights into the molecular heterogeneity of BBB disruption following TBI. EBD-labeled regions, representing areas of severe BBB disruption with high-molecular-weight extravasation, exhibited transcriptional signatures consistent with neuronal dysfunction and tissue remodeling. In contrast, NaFl-labeled regions, which remained selectively permeable to smaller molecules at later time points, were enriched in genes associated with neuroinflammation, extracellular matrix remodeling, and membrane potential regulation, reflecting a more sustained but moderate disruption of BBB integrity. This distinction may have important implications for the development and timing of therapeutic interventions, particularly for drugs that differ in molecular size and BBB penetrance. Endothelial EphA4 deletion altered these region-specific transcriptional landscapes, reducing expression of astrocyte reactivity markers such as *Gfap* and *Vim* while promoting a transcriptional profile characterized by increased expression of *Nr4a1* and *Npas4* and reduced expression of inflammatory genes including *Serpina3n* and *Slc38a1*. Together, these findings suggest that endothelial EphA4 influences both BBB permeability and the molecular response of the injured neurovascular unit following TBI.

Consistent with the improved BBB phenotype observed in KO mice, *Srf* expression was significantly increased in the KO cortex relative to WT injured tissue. *Srf* encodes Serum Response Factor (SRF), a transcription factor that plays a central role in regulating cytoskeletal dynamics, endothelial stability, and blood-brain barrier integrity. This observation is particularly noteworthy because Wnt signaling can converge on SRF-dependent pathways that promote cytoskeletal reorganization and stabilization of endothelial junctions. Together with the increased expression of BBB-associated genes (*Cldn1*,* Ocln*,* Mfsd2a*,* Tjp2*, and *Tjp3*) and enhanced Wnt/β-catenin signaling observed in KO mice, these findings suggest that endothelial EphA4 deletion promotes a transcriptional program that favors vascular stabilization following TBI. Although increased expression of BBB-associated transcripts was accompanied by reduced vascular permeability, future studies are needed to determine whether endothelial EphA4 deletion preserves tight junction protein localization and junctional continuity at the cerebrovasculature following injury.

Importantly, spatial transcriptomics provided insights into the molecular heterogeneity of BBB dysfunction that would likely be obscured by conventional bulk tissue analyses. This approach is particularly valuable for understanding the complex and heterogeneous nature of TBI, which affects different brain regions in distinct ways and results in region-specific molecular, cellular, and tissue-level responses [37–40]. By integrating spatial transcriptomics with dual-dye labeling, we were able to associate distinct transcriptional programs with specific permeability states, providing a framework for examining how BBB dysfunction evolves across injured brain regions. A limitation of our spatial transcriptomics analysis is the small biological sample size, which reduced statistical power and may have limited our ability to detect more subtle transcriptional differences. Consequently, the spatial transcriptomic findings should be interpreted primarily as hypothesis-generating and were supported by complementary histological, permeability, and molecular validation throughout the study. An additional limitation of the Visium platform is that each 55-µm capture spot contains transcripts derived from multiple cell types, including endothelial cells, astrocytes, microglia, and neurons. Consequently, the observed transcriptional changes cannot be definitively assigned to individual cellular populations. In addition to endothelial cells, pericytes and astrocytes play critical roles in maintaining BBB integrity. Whether endothelial EphA4 deletion influences pericyte coverage, astrocytic end-foot organization, AQP4 localization, or other components of the neurovascular unit remains to be determined. Future studies incorporating higher-resolution spatial approaches and computational deconvolution strategies will be necessary to determine how endothelial EphA4 deletion influences neighboring neurovascular and immune cell populations following injury.

The dual-tracer approach revealed distinct temporal dynamics of BBB permeability following CCI injury, with EBD extravasation largely resolving by 7 dpi, whereas NaFl extravasation persisted through 7 dpi and was not fully excluded until 14 dpi. These findings demonstrate that restoration of permeability to circulating tracers occurs in a size-dependent manner following CCI injury, with recovery of barrier restriction to large macromolecules preceding recovery of permeability restrictions to smaller solutes. Consistent with this observation, IgG deposition remained detectable for up to one week following injury, likely reflecting the persistence of previously extravasated endogenous proteins within the injured parenchyma. Notably, endothelial EphA4 deletion significantly reduced NaFl extravasation at 4 and 7 dpi, suggesting that loss of EphA4 attenuates vascular permeability and promotes barrier stabilization during the subacute phase of injury. Our findings further demonstrate that BBB disruption extends beyond the primary cortical lesion into the hippocampus, a region critically involved in learning and memory. Whether persistent vascular dysfunction within hippocampal circuits contributes directly to long-term neurocognitive deficits following TBI remains incompletely understood. However, chronic accumulation of blood-derived proteins, including fibrinogen, has been implicated in sustained neuroinflammation, synaptic dysfunction, and cognitive decline following CNS injury [41–48]. Importantly, BBB disruption following TBI is strongly associated with worse neurological outcomes and cognitive impairment in both experimental models and clinical populations [49, 50].

Maintenance of endothelial barrier integrity requires coordinated regulation of tight junctions, adherens junctions, and transcellular transport pathways. Consistent with the improved BBB phenotype observed in EphA4-deficient mice, spatial transcriptomic analysis revealed increased expression of multiple genes critical for BBB structure and function, including *Tjp2*,* Tjp3*,* Cdh5*,* Ocln*, and *Cldn1*. In addition, expression of *Mfsd2a*, a key suppressor of endothelial transcytosis and regulator of BBB integrity, was significantly elevated in the KO cortex. We also observed increased expression of *Ppp2r2a*, a phosphatase regulatory subunit implicated in tight junction organization and barrier maintenance. Collectively, these findings suggest that endothelial EphA4 deletion promotes a transcriptional program favoring vascular stabilization and restoration of BBB integrity following injury.

Beyond vascular-associated genes, EphA4 deletion was accompanied by increased expression of the injury-responsive transcription factors *Npas4* and *Nr4a1*, both of which have been implicated in neuronal survival, activity-dependent plasticity, and recovery following CNS injury [29, 30, 51]. Conversely, expression of the metallothionein family members *Mt1*,* Mt2*, and *Mt3* was reduced. Notably, *Mt1* has been reported to negatively regulate Wnt/β-catenin signaling by limiting β-catenin nuclear translocation [52], raising the possibility that reduced metallothionein expression may contribute to the enhanced Wnt signaling and BBB stabilization observed in EphA4-deficient mice.

Wnt/β-catenin signaling is a well-established regulator of BBB development and maintenance and has been implicated in vascular barrier restoration following CNS injury through its ability to promote endothelial stability, suppress transcytosis, and enhance expression of tight junction proteins [35, 53, 54]. Consistent with these functions, spatial transcriptomic analysis identified enrichment of multiple Wnt-related pathways and increased expression of Wnt signaling components in the injured cortex of EphA4-deficient mice. This molecular signature was accompanied by increased expression of BBB-associated genes, elevated vascular β-catenin expression, and improved barrier integrity. Importantly, pharmacological inhibition of Fzd4 signaling abolished the protective phenotype observed in EphA4-deficient mice, whereas activation of endothelial Wnt signaling using the Fzd4 agonist Fzm1.8 reduced lesion volume and attenuated BBB disruption following CCI injury. Together, these findings identify Wnt/β-catenin signaling as a critical downstream mediator of endothelial EphA4 function and suggest that EphA4 acts as a negative regulator of Wnt-dependent regulation of vascular permeability following TBI.

One limitation of the present study is that Wnt-modulating compounds were administered only during the acute phase of injury. These experiments were designed to establish a mechanistic link between endothelial EphA4 and Wnt signaling rather than define an optimal therapeutic window. Future studies will investigate how EphA4 regulates downstream Wnt signaling pathways and whether delayed activation of endothelial Wnt signaling promotes sustained restoration of vascular barrier properties during the subacute and chronic stages of injury, including preservation of tight junction protein organization, endothelial transcytosis, and neurovascular unit interactions. Additional Wnt-based therapeutics have demonstrated efficacy in protecting the blood-retinal barrier and preserving BBB integrity following ischemic stroke [55], further supporting the translational potential of targeting this pathway after neurovascular injury. An additional limitation of the present study is that the assessment of BBB integrity relied primarily on complementary permeability tracers spanning a range of molecular sizes (NaFl, Evans blue, and endogenous IgG). These assays provide functional evidence of vascular permeability but do not independently establish complete restoration of all structural and physiological properties of the BBB. While reduced tracer extravasation is consistent with improved barrier function, additional analyses examining endothelial tight-junction organization, transcellular transport, endothelial ultrastructure, and neurovascular unit interactions will be required to determine the precise mechanisms underlying the improved vascular phenotype observed following endothelial EphA4 deletion. Thus, our findings should be interpreted as evidence of attenuation of vascular permeability and barrier stabilization, rather than definitive restoration of all aspects of BBB function.

In summary, this study identifies endothelial EphA4 as a negative regulator of vascular barrier function following TBI and establishes Wnt/β-catenin signaling as a critical downstream pathway mediating vascular recovery. By integrating dual-dye labeling with spatial transcriptomics, we demonstrate that regions exhibiting distinct levels of BBB disruption possess unique molecular signatures, with EBD-positive regions enriched for vascular injury, tissue remodeling, and neuronal stress responses, whereas NaFl-positive regions exhibited prolonged but more selective BBB compromise. Endothelial EphA4 deletion reduced vascular tracer extravasation and was associated with increased expression of BBB-associated and neuroprotective genes. Furthermore, our findings highlight the therapeutic potential of activating Wnt signaling to reduce vascular permeability following TBI, as pharmacological modulation of the Wnt/Fzd4 pathway reduced lesion volume and attenuated vascular tracer extravasation following injury. Future studies should explore the translational potential of Wnt agonists for TBI therapy and further elucidate the molecular interplay between EphA4 and Wnt signaling within the neurovascular unit.

## Methods

### Animals

All mice were housed and bred in an AAALAC-accredited facility with a 12-hour light-dark cycle, and food and water were provided ad libitum. For the experiments, 7–12 male wildtype floxed *EphA4*^*f/f*^ or *EphA4*^*f/f*^*/VE-Cadherin-Cre*^*ERT2*^ mice on the CD1 background, aged 8–10 weeks, were used. All procedures followed the NIH Guide for the Care and Use of Laboratory Animals and were approved by the Virginia Tech Institutional Animal Care and Use Committee (IACUC; #24–041) and the Virginia-Maryland College of Veterinary Medicine. *Sex as a biological variable*: Male mice were used to minimize variability from hormonal fluctuations associated with the female estrous cycle, which could affect vascular and immune responses. This choice improves reproducibility and aligns with prior studies. Future work will include both sexes to assess sex-specific effects. EphA4 knockout experiments used *EphA4*^*fl/fl*^ (WT) and *EphA4*^*fl/fl*^*/VECadherin-Cre*^*ERT2*^ (KO) were bred on CD1 background for a minimum of 10 backcrosses. Adult male *EphA4*^*f/f*^ and *EphA4* ^*f/f*^*/VE-Cadherin-Cre*^*ERT2*^ mice at 8 weeks of age received intraperitoneal injections of tamoxifen (Sigma Aldrich; St. Louis, MO) at a dose of 50 mg/kg for five consecutive days. Two weeks after tamoxifen administration, genotyping was performed to verify Cre expression and EphA4 flox excision, and CCI injury was induced. The following primers were used for genotyping: FA4FLOX2: TAATTGTAATCAGTGGGCGGGC; F10718flox: GCA CAC TTA GCA ATT CAG TGT GGG; R10719flox: CCT GCA AAT TAA GGG CAG GAA GAG; oIMR1084(Cre3): GCG GTC TGG CAG TAA AAA CTA TC; oIMR1085 (Cre4): GTG AAA CAG CAT TGC TGT CAC TT.

### Controlled cortical impact (CCI) injury

Male mice were anesthetized with a pre-mixed ketamine (100 mg/kg) and xylazine (10 mg/kg) cocktail via subcutaneous injection and positioned in a stereotaxic frame, and CCI was performed as previously described [56] using Φ = 3-mm round tip connected to an eCCI-6.3 device (Custom Design & Fabrication, LLC) at − 2.0 mm M/L and 1.5 mm A/P using a velocity of 5.0 m/s, depth of 1.8 mm *±* 0.05 mm, and 150ms impact duration. Sham animals underwent craniotomy only. After 5–10 min after CCI injury, mice received a bolus injection of 10 µg/g of Fzm1.8, 10 µg/g of Fzm1, or vehicle via i.v. tail vein injection (MedChemExpress, Monmouth Junction, NJ, Fzm1.8: HY-117163, Fzm1: HY-116553). Each animal was coded, and the experimenter was blinded from group and experimental conditions. A total of 88 mice were used for this study, with the overall mortality rate of the procedure being 3.40% (3 of 91 mice). Therefore, 88 were included in the data presented within this publication. Inclusion criteria consisted of ensuring body weights falling between 30 and 50 g. Exclusion criteria included reopening of incisions, unusual behavior from the animal, poor i.v. tail vein injections of drugs or dyes, and insufficient or severe CCI injury due to user error. Outliers were determined using FDR.

### Blood-brain barrier permeability analysis

We measured BBB as previously described [57]. Briefly, a 2% sterile Evans Blue Dye (EB, Sigma E2129) and 20% Sodium Fluorescein (NaFl, F1272) solution was i.v. tail vein injected (4 mL/kg of body weight), and one-hour post-injection, the brains were perfused with 4% PFA, then post-fixed for 4 h in PFA. Serial coronal sections were rinsed with 1X PBS, then blocked in 2% cold water fish skin gelatin (Sigma, Inc.) and 0.2% Triton-X100 for 30 min. Slides were incubated with a secondary antibody for IgG staining and mounted in media containing DAPI counterstain (SouthernBiotech, Birmingham, AL). The secondaries used were all raised in donkey (Thermo Fisher Scientific, USA). Mounted tissue sections were imaged on a Nikon A1 confocal microscope using a 4x objective. Volume of extravasation (mm^3^) in the ipsilateral and contralateral cortex was assessed using the Cavalieri method with StereoInvestigator software (MicroBrightField, Williston, VT, USA) on an upright Olympus BX51TRF motorized microscope (Olympus America, Center Valley, PA, USA). The analysis was performed blindly using the confocal images of five serial coronal sections spaced 15 slices apart (− 1.1 to − 2.9 mm A/P). The data are presented as the volume of tissue damage normalized by the contralateral cortex or hippocampus (mm^3^).

### Lesion volume analysis

Lesion volume (mm^3^) in the ipsilateral cortex was assessed using the Cavalieri method with StereoInvestigator software (MicroBrightField, Williston, VT, USA) on an upright Olympus BX51TRF motorized microscope (Olympus America, Center Valley, PA, USA), as described previously (18, 32). The analysis was performed blindly using five serial coronal sections spaced 15 slices apart (− 1.1 to − 2.9 mm A/P). The data are presented as the volume of tissue damage (mm^3^).

### Visium spatial gene expression

Visium spatial transcriptomic analysis was performed as described by 10x Genomics (https://10xgenomics.com/). WT and KO mice in biological duplicates were i.v. tail vein injected with 2% EBD/20% NaFl, and the animals were perfused via the heart as described previously. Brains were frozen in optimal cutting temperature (OCT) embedding medium (Fisher Healthcare, 4585) in a cryomold. The OCT-embedded tissue blocks were stored at − 80 °C until cryosectioning. Mouse brains were cryosectioned at 20 μm and placed directly onto charged slides recommended by 10x Genomics. mRNA library preparation and sequencing using Illumina TruSeq by Medgenome (Foster City, CA).

### Spatial gene expression data analysis

Reads from several capture areas (each corresponding to injured tissue, uninjured tissue, or dye regions) were aligned to the mouse reference genome (mm10-2020-A) using Space Ranger v1.3.1 (10x Genomics). The immunofluorescence images and manual alignment files were also used as input. An average of 4,981 spots under tissue per capture area (19,924 total spots) were read and processed using Partek (San Diego, CA, an Illumina company ©). High-quality spots were selected for further analysis: cells with fewer than 200 unique feature counts and hemoglobin genes with over 30% were filtered out. After spot quality control, the dataset contained 18,961 spots under tissue with 13,186 genes detected. Median ratio was used for data normalization. Linear dimensional reduction analysis was performed with PCA (1:10 PCA dimensions). Spots gene expression and clusters were visualized within UMAP projections. Regions of interest were manually selected with the interactive contour tool, and differential expression was identified using DeSeq2. Differentially expressed genes (DEGs) were identified based on Benjamini-Hochberg-corrected *p*-value. Gene Ontology (GO) analysis was performed using DAVID [58, 59]. We report enrichment figures focusing on biologically interpretable pathways. Therefore, the top five most significant terms were selected for figure display, except for two involving “muscle system process” and “muscle contraction”.

### Statistical analysis

Data are presented as the standard error of the mean (SEM) and graphed using GraphPad Prism, version 10 (GraphPad Software, Inc., San Diego, CA). Comparison between two groups was performed using Student’s two-tailed t-test. Multiple comparisons between three or more groups using one-way ANOVA with post-hoc tests, and analysis across time points were performed using repeated-measures two-way ANOVA with post-hoc tests.

## Supplementary Information

Below is the link to the electronic supplementary material.

Supplementary Material 1.

## Data Availability

The datasets analyzed during the study are available from the corresponding author upon reasonable request.

## Abbreviations

TBI: Traumatic brain injury
CCI: Controlled cortical impact
KO: Knockout
WT: Inducible wild type
i.v.: Intravenous
dpi: Day(s) post-injury
UMAP: Uniform manifold approximation and projection

## References

[1] Haarbauer-Krupa J et al (2021) Epidemiology of chronic effects of traumatic brain injury. J Neurotrauma 38(23):3235–3247 10.1089/neu.2021.0062 PMC9122127 33947273

[2] Khatri N et al (2021) The complexity of secondary cascade consequent to traumatic brain injury: pathobiology and potential treatments. Curr Neuropharmacol 19(11):1984–2011 10.2174/1570159X19666210215123914 PMC9185786 33588734

[3] Stark R, Grzelak M, Hadfield J (2019) RNA sequencing: the teenage years. Nat Rev Genet 20(11):631–656 10.1038/s41576-019-0150-2 31341269

[4] Tang F et al (2009) mRNA-Seq whole-transcriptome analysis of a single cell. Nat Methods 6(5):377–382 10.1038/nmeth.1315 19349980

[5] Arneson D et al (2018) Single cell molecular alterations reveal target cells and pathways of concussive brain injury. Nat Commun 9(1):3894 10.1038/s41467-018-06222-0 PMC6156584 30254269

[6] Garza R et al (2023) Single-cell transcriptomics of human traumatic brain injury reveals activation of endogenous retroviruses in oligodendroglia. Cell Rep 42(11):113395 10.1016/j.celrep.2023.113395 37967557

[7] Qiu X et al (2023) Single-cell RNA-sequencing analysis reveals enhanced non-canonical neurotrophic factor signaling in the subacute phase of traumatic brain injury. CNS Neurosci Ther 29(11):3446–3459 10.1111/cns.14278 PMC10580338 37269057

[8] Kounelis-Wuillaume SK et al (2026) A spatial gene expression signature of the mouse brain post-injury at the focal point of contusion. J Neurotrauma 43(5–6):461–484 10.1177/08977151251390528 41250842

[9] Sullivan GM et al (2026) Mapping the secondary response to traumatic brain injury using spatial transcriptomics shows acute 4-aminopyridine treatment mitigates axonal and molecular pathology. Acta Neuropathol Commun 14(1):36 10.1186/s40478-025-02219-1 PMC12882205 41508250

[10] Swaro A et al (2025) Widespread and cell-type-specific transcriptomic reorganization following mild traumatic brain injury. Cell Rep 44(6):115795 10.1016/j.celrep.2025.115795 40478731

[11] Yen C, Lin CL, Chiang MC (2023) Exploring the frontiers of neuroimaging: a review of recent advances in understanding brain functioning and disorders. Life (Basel) 13(7):1472 10.3390/life13071472 PMC10381462 37511847

[12] Obenaus A, Badaut J (2022) Role of the non-invasive imaging techniques in monitoring and understanding the evolution of brain edema. J Neurosci Res 100(5):1191–1200 10.1002/jnr.24837 34048088

[13] Ya D et al (2023) Application of spatial transcriptome technologies to neurological diseases. Front Cell Dev Biol 11:1142923 10.3389/fcell.2023.1142923 PMC10020196 36936681

[14] Yao L et al (2018) Evans blue dye: a revisit of its applications in biomedicine. Contrast Media Mol Imaging 2018:p7628037 10.1155/2018/7628037 PMC5937594 29849513

[15] Grimbleby FH, Ntailianas HA (1961) Binding of trichloroacetic acid by protein. Nature 189:835–836 10.1038/189835a0 13708701

[16] Duan H, Cheng T, Cheng H (2023) Spatially resolved transcriptomics: advances and applications. Blood Sci 5(1):1–14 10.1097/BS9.0000000000000141 PMC9891446 36742187

[17] Zheng P et al (2023) Integrated spatial transcriptome and metabolism study reveals metabolic heterogeneity in human injured brain. Cell Rep Med 4(6):101057 10.1016/j.xcrm.2023.101057 PMC10313933 37263268

[18] Cash A et al (2023) Endothelial deletion of EPH receptor A4 alters single-cell profile and Tie2/Akap12 signaling to preserve blood-brain barrier integrity. Proc Natl Acad Sci U S A 120(41):e2204700120 10.1073/pnas.2204700120 PMC10576133 37796990

[19] Frugier T et al (2012) Expression and activation of EphA4 in the human brain after traumatic injury. J Neuropathol Exp Neurol 71(3):242–250 10.1097/NEN.0b013e3182496149 22318127

[20] Xu NJ, Henkemeyer M (2012) Ephrin reverse signaling in axon guidance and synaptogenesis. Semin Cell Dev Biol 23(1):58–64 10.1016/j.semcdb.2011.10.024 PMC3288821 22044884

[21] Malik VA, Benedetto BD (2018) The blood-brain barrier and the EphR/Ephrin system: perspectives on a link between neurovascular and neuropsychiatric disorders. Front Mol Neurosci 11:127 10.3389/fnmol.2018.00127 PMC5906525 29706868

[22] Baskaya MK et al (1997) The biphasic opening of the blood-brain barrier in the cortex and hippocampus after traumatic brain injury in rats. Neurosci Lett 226(1):33–36 10.1016/s0304-3940(97)00239-5 9153635

[23] Beamer M et al (2016) Primary blast injury causes cognitive impairments and hippocampal circuit alterations. Exp Neurol 283(Pt A):16–28 10.1016/j.expneurol.2016.05.025 PMC5062598 27246999

[24] Rola R et al (2006) Alterations in hippocampal neurogenesis following traumatic brain injury in mice. Exp Neurol 202(1):189–199 10.1016/j.expneurol.2006.05.034 16876159

[25] Zhang Z et al (2020) Pediatric traumatic brain injury causes long-term deficits in adult hippocampal neurogenesis and cognition. J Neurotrauma 37(14):1656–1667 10.1089/neu.2019.6894 32079496

[26] Dragunow M, Robertson HA (1988) Brain injury induces c-fos protein(s) in nerve and glial-like cells in adult mammalian brain. Brain Res 455(2):295–299 10.1016/0006-8993(88)90088-1 3135922

[27] Pekny M, Pekna M (2004) Astrocyte intermediate filaments in CNS pathologies and regeneration. J Pathol 204(4):428–437 10.1002/path.1645 15495269

[28] Matzilevich DA et al (2002) High-density microarray analysis of hippocampal gene expression following experimental brain injury. J Neurosci Res 67(5):646–663 10.1002/jnr.10157 11891777

[29] Choy FC et al (2016) Reduction of the neuroprotective transcription factor Npas4 results in increased neuronal necrosis, inflammation and brain lesion size following ischaemia. J Cereb Blood Flow Metab 36(8):1449–1463 10.1177/0271678X15606146 PMC4976743 26661154

[30] Wu X et al (2019) NDP-MSH binding melanocortin-1 receptor ameliorates neuroinflammation and BBB disruption through CREB/Nr4a1/NF-kappaB pathway after intracerebral hemorrhage in mice. J Neuroinflammation 16(1):192 10.1186/s12974-019-1591-4 PMC6816206 31660977

[31] Weinl C et al (2015) Endothelial depletion of murine SRF/MRTF provokes intracerebral hemorrhagic stroke. Proc Natl Acad Sci U S A 112(32):9914–9919 10.1073/pnas.1509047112 PMC4538672 26221020

[32] Goffigan-Holmes J et al (2019) Calbindin-1 expression in the hippocampus following neonatal hypoxia-ischemia and therapeutic hypothermia and deficits in spatial memory. Dev Neurosci 40:1–15 10.1159/000497056 PMC6742590 30861522

[33] Zhang L et al (2023) A Frizzled4-LRP5 agonist promotes blood-retina barrier function by inducing a Norrin-like transcriptional response. iScience 26(8):107415 10.1016/j.isci.2023.107415 PMC10407957 37559903

[34] Wang Z et al (2020) Wnt signaling activates MFSD2A to suppress vascular endothelial transcytosis and maintain blood-retinal barrier. Sci Adv 6(35):eaba7457 10.1126/sciadv.aba7457 PMC7455181 32923627

[35] Laksitorini MD et al (2019) Modulation of Wnt/beta-catenin signaling promotes blood-brain barrier phenotype in cultured brain endothelial cells. Sci Rep 9(1):19718 10.1038/s41598-019-56075-w PMC6928218 31873116

[36] Riccio G et al (2018) A negative allosteric modulator of WNT receptor frizzled 4 switches into an allosteric agonist. Biochemistry 57(5):839–851 10.1021/acs.biochem.7b01087 29293331

[37] Piwecka M, Rajewsky N, Rybak-Wolf A (2023) Single-cell and spatial transcriptomics: deciphering brain complexity in health and disease. Nat Rev Neurol 19(6):346–362 10.1038/s41582-023-00809-y PMC10191412 37198436

[38] McAllister TW (2011) Neurobiological consequences of traumatic brain injury. Dialogues Clin Neurosci 13(3):287–300 10.31887/DCNS.2011.13.2/tmcallister PMC3182015 22033563

[39] Arneson D et al (2022) Systems spatiotemporal dynamics of traumatic brain injury at single-cell resolution reveals humanin as a therapeutic target. Cell Mol Life Sci 79(9):480 10.1007/s00018-022-04495-9 PMC9372016 35951114

[40] Jha RM et al (2024) A single-cell atlas deconstructs heterogeneity across multiple models in murine traumatic brain injury and identifies novel cell-specific targets. Neuron 112(18):3069–3088e4 10.1016/j.neuron.2024.06.021 PMC11578855 39019041

[41] Scoville WB, Milner B (2000) Loss of recent memory after bilateral hippocampal lesions. 1957. J Neuropsychiatry Clin Neurosci 12(1):103–113 10.1176/jnp.12.1.103 10678523

[42] Milner B (1972) Disorders of learning and memory after temporal lobe lesions in man. Clin Neurosurg 19:421–446 10.1093/neurosurgery/19.cn_suppl_1.421 4637561

[43] Rempel-Clower NL et al (1996) Three cases of enduring memory impairment after bilateral damage limited to the hippocampal formation. J Neurosci 16(16):5233–5255 10.1523/JNEUROSCI.16-16-05233.1996 PMC6579309 8756452

[44] Iggena D et al (2023) Multisensory input modulates memory-guided spatial navigation in humans. Commun Biol 6(1):1167 10.1038/s42003-023-05522-6 PMC10646091 37963986

[45] Arciniegas DB, Held K, Wagner P (2002) Cognitive impairment following traumatic brain injury. Curr Treat Options Neurol 4(1):43–57 10.1007/s11940-002-0004-6 11734103

[46] Skelton RW et al (2000) Humans with traumatic brain injuries show place-learning deficits in computer-generated virtual space. J Clin Exp Neuropsychol 22(2):157–175 10.1076/1380-3395(200004)22:2;1-1;FT157 10779831

[47] Sulimai N, Lominadze D (2020) Fibrinogen and neuroinflammation during traumatic brain injury. Mol Neurobiol 57(11):4692–4703 10.1007/s12035-020-02012-2 PMC7530020 32776201

[48] Patel S et al (2024) From blood to brain: exploring the role of fibrinogen in the pathophysiology of depression and other neurological disorders. Int Immunopharmacol 143(Pt 1):113326 10.1016/j.intimp.2024.113326 39388892

[49] Che J et al (2024) Blood-brain barrier disruption: a culprit of cognitive decline? Fluids Barriers CNS 21(1):63 10.1186/s12987-024-00563-3 PMC11305076 39113115

[50] Yang J et al (2021) Vitamin D3 supplement attenuates blood-brain barrier disruption and cognitive impairments in a rat model of traumatic brain injury. Neuromolecular Med 23(4):491–499 10.1007/s12017-021-08649-z 33616826

[51] Rothe T et al (2017) The nuclear receptor Nr4a1 acts as a microglia rheostat and serves as a therapeutic target in autoimmune-driven central nervous system inflammation. J Immunol 198(10):3878–3885 10.4049/jimmunol.1600638 PMC5798579 28411187

[52] Dai H et al (2021) Metallothionein 1: a new spotlight on inflammatory diseases. Front Immunol 12:739918 10.3389/fimmu.2021.739918 PMC8602684 34804020

[53] Liebner S et al (2008) Wnt/β-catenin signaling controls development of the blood-brain barrier. J Cell Biol 183(3):409–417 10.1083/jcb.200806024 PMC2575783 18955553

[54] Hussain B et al (2022) Endothelial beta-Catenin deficiency causes blood-brain barrier breakdown via enhancing the paracellular and transcellular permeability. Front Mol Neurosci 15:895429 10.3389/fnmol.2022.895429 PMC9125181 35615065

[55] Ding J et al (2023) Therapeutic blood-brain barrier modulation and stroke treatment by a bioengineered FZD(4)-selective WNT surrogate in mice. Nat Commun 14(1):2947 10.1038/s41467-023-37689-1 PMC10238527 37268690

[56] Kowalski EA et al (2019) Peripheral loss of EphA4 ameliorates TBI-induced neuroinflammation and tissue damage. J Neuroinflammation 16(1):210 10.1186/s12974-019-1605-2 PMC6844068 31711546

[57] Brickler TR et al (2018) Angiopoietin/Tie2 axis regulates the age-at-injury cerebrovascular response to traumatic brain injury. J Neurosci 38(45):9618–9634 10.1523/JNEUROSCI.0914-18.2018 PMC6222064 30242049

[58] Huang da W, Sherman BT, Lempicki RA (2009) Systematic and integrative analysis of large gene lists using DAVID bioinformatics resources. Nat Protoc 4(1):44–57 10.1038/nprot.2008.211 19131956

[59] Huang da W, Sherman BT, Lempicki RA (2009) Bioinformatics enrichment tools: paths toward the comprehensive functional analysis of large gene lists. Nucleic Acids Res 37(1):1–13 10.1093/nar/gkn923 PMC2615629 19033363

