# Supplementary material for: Spatial transcriptomics and dual tracer mapping reveal Wnt-driven attenuation of blood-brain barrier permeability Induced by endothelial EphA4 deficiency
Source: Acta Neuropathol Commun. 2026 Aug 6;14:230. doi: 10.1186/s40478-026-02394-9 (PMC13741444; doi:10.1186/s40478-026-02394-9)
Supplement: Supplementary file 1 [file 40478_2026_2394_MOESM1_ESM.docx]

 **Supplemental Figure 1.** **Time-resolved analysis of BBB disruption using dual dye-labeling following CCI injury.** (A) Experimental timeline illustrating tail vein injection (TVI) with Evans Blue Dye (EBD) and Sodium Fluorescein (NaFl) 1 hour before perfusion at 1, 4, and 7 days post-CCI injury (dpi). Tissue was collected via cryosectioning and analyzed using confocal imaging for volumetric assessment. (B–E) Representative coronal brain sections showing extravasation of EBD and NaFl at different anteroposterior levels relative to bregma at 1dpi. (B) Naïve brain with intact BBB showing no tracer extravasation. (F–H) Confocal images of injured cortical regions at 1 dpi (F), 4 dpi (G), and 7 dpi (H). Merged images (top row) show colocalization of IgG (red), EBD (magenta), and NaFl (green), indicating progressive BBB repair over time. The middle rows show individual channels for IgG and EBD, highlighting the severity of extravasation, while the bottom row (NaFl) shows small-molecule leakage. Scale bars: 1 mm. (I-K) Representative confocal images of NaFl extravasation at 14dpi. (I) Merged, (J) DAPI (blue), and (K) NaFl (green), confocal images. Scale bar = 1mm.

**Supplemental Figure 2: Volumetric analysis of EBD and NaFl extravasation into the injured cortex.** (A) Ipsilateral and contralateral extravasation of EBD and NaFl at 1dpi in the WT and KO injured cortex. (B) Ipsilateral and contralateral extravasation of EBD and NaFl at 4dpi in the WT and KO injured cortex. (C) Ipsilateral and contralateral extravasation of EBD and NaFl at 7dpi in the WT and KO injured cortex. Statistical significance was determined using two-way ANOVA, Tukey post-hoc. Data presented as mean ± SEM; **p < 0.01, ***p<0.001, ****p < 0.0001, ns = not significant. n=8/group.

**Supplemental Figure 3: EC-specific deletion of EphA4 reduces hippocampal dye extravasation following CCI injury** Representative confocal images of WT and KO injured tissue. Insets show hippocampal regions with merged dyes (A.1, B.1), NaFl, green (A.2, B.2), IgG, red (A.3, B.3), and EBD, magenta (A.4, B.4) White dotted lines represent the perimeter of the cortical injury site. (C–C.2) Quantification of hippocampal temporal extravasation of NaFl (C), EBD (C.1), and IgG (C.2) in WT and KO mice. Statistical significance was determined using two-way ANOVA; Bonferroni post-hoc. Data presented as mean ± SEM; **p < 0.01, ***p<0.001, ****p < 0.0001, ns = not significant. Scale bar = 1mm. n=8-10.
